# The influence of time in captivity, food intake and acute trauma on blood analytes of juvenile Steller sea lions, *Eumetopias jubatus*

**DOI:** 10.1093/conphys/cov008

**Published:** 2015-03-13

**Authors:** John P. Skinner, Pam A. Tuomi, Jo-Ann E. Mellish

**Affiliations:** 1 Alaska SeaLife Center, Box 1329, Seward, AK 99664, USA; 2 School of Fisheries and Ocean Sciences, University of Alaska Fairbanks, Fairbanks, AK 99775, USA

**Keywords:** Biochemistry, capture effects, *Eumetopias jubatus*, fasting, haematology, wounding

## Abstract

The Steller sea lion, *Eumetopias jubatus*, has experienced regionally divergent population trends over recent decades. One potential mechanism for this disparity is that local factors cause reduced health and, therefore, reduced survival of individuals. The use of blood parameters to assess sea lion health may help to identify whether malnutrition, disease and stress are important drivers of current trends, but such assessments require species-specific knowledge of how parameters respond to various health challenges. We used principal components analysis to identify which key blood parameters (principal analytes) best described changes in health for temporarily captive juvenile Steller sea lions in known conditions. Generalized additive mixed models were used to estimate the changes in principal analytes with food intake, time in captivity and acute trauma associated with hot-iron branding and transmitter implant surgery. Of the 17 blood parameters examined, physiological changes for juvenile sea lions were best described using the following six principal analytes: red blood cell counts, white blood cell counts, globulin, platelets, glucose and total bilirubin. The white blood cell counts and total bilirubin declined over time in captivity, whereas globulin increased. Elevated red blood cell counts, white blood cell counts and total bilirubin and reduced globulin values were associated with lower food intake. After branding, white blood cell counts were elevated for the first 30 days, while globulin and platelets were elevated for the first 15 days only. After implant surgery, red blood cell counts and globulin remained elevated for 30 days, while white blood cell counts remained elevated during the first 15 days only. Glucose was unassociated with the factors we studied. These results were used to provide expected ranges for principal analytes at different levels of food intake and in response to the physical challenges of branding and implant surgery. These results provide a more detailed reference for future evaluations of health-related assessments.

## Abstract (precis)

We identified five blood parameters associated with physiological changes experienced by wild juvenile Steller sea lions while held in temporary captivity. These key parameters could offer a way to better characterize the health status of juvenile Steller sea lion populations in the wild.

## Introduction

Steller sea lions (*Eumetopias jubatus*) in the USA have followed different trajectories across their range over the past few decades. For management purposes, the population was divided into the western and the eastern distinct population segments at 144° West longitude (Cape Suckling, AK, USA). The western distinct population segment experienced a 70% population decline during a 30 year period beginning in the 1960s (Merrick *et al*., 1995), whereas the genetically distinct eastern population remained relatively stable. Currently, declines continue in the western portion of the Aleutian Islands, but recent surveys suggest that populations are growing in the Gulf of Alaska and Southeast Alaska (Fritz *et al.*, 2013). The western distinct population segment remains listed as endangered under the Endangered Species Act, but the eastern distinct population segment, which was previously categorized as threatened, has been delisted (National Marine Fisheries Service, 2013). The underlying causes for these divergent population trends remain unclear (National Marine Fisheries Service, 2008); however, health factors have been investigated by looking at the prevalence of disease or malnutrition using haematology and clinical chemistry panels. This technique is appealing because obtaining blood is a relatively simple process and can offer clues to multiple disease, nutrition and stress modalities (Bossart *et al*., 2001; Hall *et al*., 2010). The most common approach has been to compare parameters from the western with the eastern distinct population segment. Plasma concentrations of haptoglobin, an acute phase protein, suggested potentially elevated stress levels among Steller sea lions in the Gulf of Alaska compared with those from Southeast Alaska (Zenteno-Savin *et al*., 1997). Additionally, chemistry and haematological values for pups in areas of decline compared with those from stable areas supported the suggestion that pup condition was probably not compromised in the western distinct population segment (Rea *et al*., 1998; Lander *et al.*, 2013). These comparative studies are useful for examining potential causes underlying differing population trends; however, accurate interpretation requires specific knowledge about how blood parameters respond when animals are confronted with different physiological challenges. This reference information can be difficult to attain because it requires controlled studies that are logistically impossible to implement on free-ranging individuals.

Captive research has extreme value in providing a way to study changes in blood parameters in a controlled, experimental setting. Measurement of the response of blood parameters to known stressors provides an integral understanding of the clinical and physiological relevance to changes observed in the wild. For example, metabolite profiles obtained from captive juvenile and subadult Steller sea lions revealed that food restriction significantly affects blood serum ketone bodies and blood urea nitrogen concentrations (Rea *et al*., 2009). Additionally, temporary captive research on juvenile sea lions has been used to create previously unknown age-specific baseline values for numerous standard health-screening parameters (Mellish *et al*., 2006). These values then provide a comparison for the assessment of a range of treatments, including behavioural and physiological effects of trauma caused by hot-iron branding (Mellish *et al*., 2007a) and surgical implantation of transmitters (Mellish *et al*., 2007b).

Despite the advantages of captive studies, some consideration must be given when using data from captive animals to interpret the physiology of free-ranging individuals. For instance, holding animals for any extended period could result in changes to the physiology of the individuals studied. Detailed monitoring of animals in captivity has been largely limited to harbour seal pups in rehabilitation. These studies have demonstrated significant changes in multiple haematology and clinical chemistry parameters, although some may be attributed to poor health at admission and early developmental stage (Lander *et al*., 2003; Greig *et al*., 2010). Changes in white blood cell count and serum calcium levels were reported over the course of temporary captivity in juvenile Steller sea lions that were considered a representative subsample of the local population (Mellish *et al*., 2006). Although the exact causes of these changes are not fully understood, they may relate to changes in behaviour, such as reduced exercise and restricted diving within enclosures, changes in diet and quantity of food consumed, changes in parasite exposure as a result of being kept in a cleaner environment, or elevated levels of acute or chronic stress. Additionally, some of these factors and their effects may change throughout time in captivity (e.g. Lander *et al*., 2003; Mellish *et al*., 2006). Failing to account for these effects may produce results that poorly describe the physiology of free-ranging individuals. It has been found that values for blood analytes of healthy captive individuals (i.e. ‘normals’) may be elevated or depressed relative to those of individuals residing in the wild for a wide range of mammals (e.g. harbour seals, Hasselmeier *et al*., 2008; possum, Clarke *et al*., 2013; wallabies, Robert and Schwanz, 2013). Furthermore, the effects of captive research could extend beyond the holding, because released individuals may undergo a period of readjustment of uncertain duration (e.g. diving or foraging behaviour, Thomton *et al*., 2008).

Captive research has proved useful for examining otherwise intractable study questions (e.g. the impact of switching diet, Calkins *et al*., 2013), but the approach remains somewhat limited in scope and application. For many exotic species, it remains the primary venue for the collection of any baseline information (e.g. large cats, Pospísil *et al.*, 1987). Ethical concerns prevent us from exploring those extreme factors that are likely to alter behaviour and impact near-term and extended survival in the wild. Advanced modelling techniques may provide a means to explore the effects of those conditions beyond the limitations of the study and control for captive effects, thereby improving our understanding of the physiology for free-ranging animals.

This study specifically builds on previous assessments of temporarily captive juvenile Steller sea lions, seeking to assess the varying levels and patterns of physiological response to hot-iron branding (Mellish *et al*., 2007a), surgical implantation of transmitters (Horning *et al*., 2008; Walker *et al*., 2009) and captivity itself (Mellish *et al*., 2006). The aims of this study were as follows: (i) to monitor the pattern of change in blood analytes in response to a range of physiological challenges in controlled conditions; (ii) to identify the primary analytes of each documented shift through principal components analysis; and (iii) to compare the physiological responses to the varied challenges of captivity, hot-iron branding and surgical implantation, accounting for the effect of nutritional state.

## Materials and methods

### Captivity

As a part of a long-term research programme, 54 juvenile Steller sea lions, *E. jubatus*, aged 12–24 months based on tooth eruption patterns (King *et al*., 2007) were captured in 13 groups from Prince William Sound, AK, USA, between August 2003 and May 2009. Of these, 35 sea lions from 10 groups were selected for the present study (see ‘*Definitions for variables*’). Sea lions were captured by underwater lasso (McAllister *et al*., 2001), retrieved with a skiff and transported to a larger vessel for processing. Within 3 h of capture, an initial blood sample was obtained at 5–10 min from the start of anaesthesia (see ‘*Blood sampling and treatment protocols*’). Individuals were transported to a specialized outdoor quarantine facility at the Alaska SeaLife Center in Seward, AK, USA within 48 h. Depending on the specific research objectives, individuals were held in captivity for 42–120 days before release (Mellish *et al*., 2006). Sea lions were generally fed to satiation, except immediately before handling. Daily food consumption by each sea lion was tracked by weighing the amount of fish offered and subtracting the weight of parts that remained in pools after each feeding (estimated to the nearest 0.1 kg). All procedures were carried out under National Marine Fisheries Service permits 881-1668, 881-1890 and 14335 and approved by institutional animal use and care protocols.

### Blood sampling and treatment protocols

Animals were fasted overnight before each handling event. They were initially restrained manually and then administered isoflurane gas for the duration of the procedure (AErrane; Baxter Healthcare Corporation, Deerfield, IL, USA; induction level of 5% in oxygen, reduced to 1–3% as needed). Blood was collected from the caudal plexus or hind-flipper vein at 5–10 min following induction of anaesthesia. Blood samples were analysed within 10 min using a Vetscan HMT II^®^ and VetScan chemistry analyser (Abaxis, Union City, CA, USA). After blood sampling and while the sea lion was still anaesthetized, additional procedures were performed. Of the 35 sea lions included in the study, 30 were hot branded for permanent marking using protocols outlined by Mellish *et al*. (2007a). Briefly, branding irons, each with a unique symbol or digit, were heated until cherry red and then applied to the left side of the animal for 2–4 s (Merrick *et al*., 1996). Brands generally started with an equal sign (‘=’) applied at the shoulder, followed by three digits, 10.2 cm high by 5.1 cm wide (for photographss see Mellish *et al*. 2007a), applied to the skin of the lateral thorax caudal to each preceding digit. Twenty-six branded and one unbranded individual were surgically implanted with two 115 g, cylindrically shaped (122 mm long by 42 mm diameter) life-history transmitters (LHX; Horning and Hill, 2005) using surgical techniques described by Horning *et al*. (2008). This procedure required that an 8.5–12 cm longitudinal incision be made along the ventral mid-line through the skin, linea alba and peritoneum. After implant placement, each layer was closed using synthetic absorbable sutures. Animals were allowed to recover from anaesthesia in dry holding area before release into areas with water access. The timing and order of the two treatments were not consistent across individuals, but they were always conducted sometime after the first week of captivity. The blood sampling and treatment schedule during each 2 week period of captivity is shown in Table 1.

**Table 1:** Blood sampling and treatment schedule for juvenile Steller sea lions held in captivity

Animal ID	Year	Sex	Capture mass (kg)	Duration of captivity (days)							
				0	1–15	16–30	31–45	46–60	61–75	76–90	Total
TJ002	2003	F	95	1	2	2	1	—	—	—	6
TJ004	2003	F	132	1	1	1	1	1	—	—	5
TJ017^a^	2005	M	104	1	1	1^B^	3	1	1	—	8
TJ018^a^	2005	M	98	1	1	1	1	2	1^B^	—	7
TJ019^a^	2005	F	111	1	1	1^B^	3	1	1	—	8
TJ020^a^	2005	F	107	1	1	1^B^	1	2	1	—	7
TJ022^a^*^,^*^b^	2005	F	119	1	—	1	1^L^	1	1	1^B^	6
TJ023^a^*^,^*^b^	2005	M	109	1	—	1	1	2^L^	1^B^	—	6
TJ024^b^	2006	M	153	1	—	2	2^L^	2^B^	1	—	8
TJ025^b^	2006	M	152	1	—	2	1^L^	2	1	—	7
TJ026^b^	2006	M	161	1	—	2	2^L^	1	1^B^	—	7
TJ027^b^	2006	M	167	1	—	2	2^L^	2^B^	1	—	8
TJ029	2006	M	201	1	—	1	2	1	1	—	6
TJ031	2006	M	168	1	—	1	2	1	1	—	6
TJ032	2007	M	160	1	—	2	1	1^L^	1^B^	—	6
TJ033	2007	M	134	1	—	2	1	1^L^	1^B^	—	6
TJ034	2007	M	102	1	—	2	1	1^B+L^	1	—	6
TJ035	2007	M	150	1	—	2	1	1^B+L^	1	—	6
TJ036	2007	M	99	1	—	2	1	1^B+L^	1	—	6
TJ038	2008	M	203	1	1	1	1	1^B+L^	1	—	6
TJ039	2008	M	150	1	1	1	1	1^B+L^	1	—	6
TJ040	2008	F	102	1	1	1	1	1^L^	1^B^	—	6
TJ041	2008	M	159	1	1	1	1	1^L^	1^B^	—	6
TJ043	2008	F	153	1	1	1^B^	1	1	1	1^L^	7
TJ044	2008	M	111	1	1	1^B^	1	1	1	1^L^	7
TJ045	2008	M	142	1	1	1^B^	1	1	1	1^L^	7
TJ046	2008	M	120	1	1	1^B^	1	1	1	1^L^	7
TJ047	2008	F	103	1	1	1^B^	1	1	1	1^L^	7
TJ048	2008	M	108	1	1	1^B^	1	1	1	1^L^	7
TJ050	2009	M	127	1	1	1^B^	1	1	1	1^L^	7
TJ051	2009	F	121	1	1	1^B^	1	1	2^L^	—	7
TJ052	2009	M	150	1	1	1^B^	1	1	1	1^L^	7
TJ053	2009	M	74	1	1	1^B^	1	1	1	3^L^	9
TJ054	2009	M	95	1	1	1^B^	1	1	3^L^	—	8
TJ055	2009	M	91	1	1	1^B^	1	1	3^L^	1	9

Shown here for each sea lion is year of capture, sex (F, female or M, male), mass at capture (in kilograms) and the number of blood samples obtained during each interval of time while in captivity. Superscripts indicate that the animal received a treatment during the interval. Treatments include branding (B), abdominal implant of a life-history transmitter (L) or both (B + L); ‘—’ indicates that no samples were obtained during the specified period. ^a^Branding effects previously reported by Mellish *et al*. (2007a). ^b^Implant surgery effects previously reported by Mellish *et al.* (2007b).

### Definitions for variables

Given that blood was collected for the purposes of health assessment and several concurrent research projects, few blood analytes were obtained consistently across all captive groups; therefore 35 animals were chosen for this study based on having had blood analysed for a specific set of 17 analytes more than six times during captivity. These analytes included 12 biochemical parameters [albumin (ALB; in grams per decilitre), alkaline phosphatase (ALP; in units per litre), alanine amino transferase (ALT; in units per litre), amylase (AMY; in units per litre), blood urea nitrogen (BUN; in milligrams per decilitre), calcium (CA; in milligrams per decilitre), creatinine (CRE; in milligrams per decilitre), globulin (GLOB; in grams per decilitre), glucose (GLU; in milligrams per decilitre), haemoglobin (Hb; in grams per decilitre), potassium (K; in millimoles per litre), total bilirubin (TBIL; in milligrams per decilitre) and total protein (TP; in grams per decilitre)] and four haematological values [haematocrit (HCT; expressed as a percentage), platelet count (PLT; ×10^3^ per cubic millimetre), red blood cell count (RBC; ×10^6^ per cubic millimetre) and white blood cell count (WBC; ×10^3^ per cubic millimetre)]. As the length of sea lion captivity was generally shorter than 90 days, we excluded less frequent samples obtained >90 days to balance the number of samples across animals. Each blood analyte was inspected for normality, and a logarithmic transformation was applied to RBC, WBC, ALP, PLT, AMY and ALT, while HCT was transformed by applying an arcsine square-root transformation. Time in captivity (*tCaptive*; in days) was calculated as the day of a blood sampling event minus the day of capture. Food intake (in kilograms) was estimated as the average food consumed during a 3 day period before blood sampling. Time since branding (*SinceBrand*; in days) was the number of days since branding had occurred and was treated as a factor with the following five levels: ‘no brand’ and 1–15, 16–30, 31–45 and >45 days after branding. Time since LHX implant (*SinceImplant*; in days) was the number of days since implant surgery and was treated as a factor with the following four levels: ‘no LHX’, 1–15, 16–30 and >30 days post-LHX implant. The break points for levels within each factor were chosen, in part, to equalize the number of observations within each level. Sea lions in the study were disproportionately male (26 males vs. nine females), which limited our ability to make statistical inferences about females. Furthermore, we did not expect large differences due to sex hormones in this age group; therefore, we pooled the analysis across ‘sex’.

### Trends in food intake

Sea lions may undergo a voluntary fast of varying length during the initial days of captivity (Mellish *et al*., 2006). We also noted that appetite declines following periods of vigorous eating and after LHX implant surgery. To characterize these changes, we used a generalized additive mixed model (GAMM) with an identity link (R-package ‘mgcv’, http://cran.r-project.org/web/packages/mgcv/index.html; Wood, 2006) to estimate changes in food intake (response) with respect to *tCaptive*, *SinceBrand* and *SinceImplant* (predictors). All statistical modelling was conducted using the R software package (R Core Team, 2012), and an α level of 0.05 was used to determine statistical significance. Repeated measures on an individual (subject effect) were accounted for in the model by including sea lion identifier as a random effect (intercept only). To estimate non-linear changes in food intake throughout captivity, *tCaptive* was modelled using a thin plate regression spline limited to a five degree polynomial (*k* = 6). The model with all predictor variables was validated against the null model (random effect only) using a log-likelihood ratio test (McCullagh and Nelder, 1989), and model goodness of fit was assessed by inspection of residual and Q-Q plots. As we hypothesized that treatments would immediately affect feeding behaviour (as opposed to a delayed effect of >14 days), we used linear contrasts between the period with no treatment (‘no brand’ or ‘no LHX’) and 1–15 days to determine whether the treatment significantly altered feeding behaviour. The effects of significant predictor variables were depicted using partial residual plots (Wood, 2006).

### Principal analytes

Principal components analysis (PCA; package ‘psych’, Revelle, 2013) was performed on the 17 analyte values obtained from blood samples collected during all handling events except the initial capture (*n* = 203). As a result of the different scales among analytes, the correlation matrix was used in performing the analysis. This analysis allowed the variances observed in otherwise partly correlated blood analytes to be explained using a smaller number of uncorrelated variables called principal components (PCs). We used a rule that PCs with eigenvalues > 1 were retained for the final solution produced using a ‘varimax’ rotation. The standardized loading scores produced by the PCA were used to evaluate which blood analytes were most associated with each PC. The sign of these scores indicated whether associations were negative or positive, while the magnitude (0–1) indicated the strength of association. Blood analytes with loading scores >0.40 or <−0.40 were considered to be highly associated with, and therefore important for explaining the physiological processes driving the variance of, a PC (McGarigal *et al*., 2000). Additionally, we used the communality score ($\hat{C}$), a measure of the amount of analyte variance explained by all PCs, to determine how well the analysis captured changes in a particular analyte. For each PC, the analyte with the highest absolute standardized loading score was chosen to represent the process described by that PC (i.e. principal analyte) and was modelled as the response value for examining factors potentially influencing sea lion physiology while in captivity.

### Modelling factors influencing principal analytes

Similar to the method described above, GAMMs with an identity link were used to evaluate how principal analytes during all sampling events, except the initial capture sample, were influenced by the following predictor variables: *tCaptive* and food intake, modelled as thin plate regression splines limited to a second degree polynomial (*k* = 3); *SinceBrand* and *SinceImplant* as factors; and sea lion identifier as a random effect. For each principal analyte, a global model was fitted and validated against the null model using a log-likelihood ratio test, and residuals were inspected to determine the model fit. Predictors originally modelled as splines but later found to have estimated degrees of freedom equal to one (edf = 1) in the global model were reanalysed as linear predictors, and models were rerun. A candidate set of models was developed for each principal analyte using all combinations of the main effects and a null model (16 models in total). Model-averaging methods (Burnham and Anderson, 2002) were used to analyse each set of candidate models, ranked by second-order Akaike information criterion, and results were used to calculate parameter estimates, unconditional standard errors and 95% confidence intervals for each predictor using the R package, ‘MuMIn’ (Barton, 2013). A best-fit model was produced for each principal analyte by removing all non-significant predictor variables (i.e. model-average parameter estimates with 95% confidence interval that included zero) and used to calculate an adjusted coefficient of determination (adjusted *r*^2^). The fixed effects for best-fit models were used to produce predicted values for analytes at different levels of food intake, with and without the effects of minor trauma resulting from branding and LHX implant surgery. Food intake used in these predictions was categorized as follows: ‘fasted’, 0 kg/day; ‘moderate’, the median intake rate (6.4 kg/day); and ‘high’, the mean of maximal rates (9.3 kg/day) calculated using all captive observations. Model predictions for the effects of recent trauma (<15 days) were produced assuming moderate food intake (6.4 kg/day).

## Results

### Factors influencing food intake

**Figure 1:**
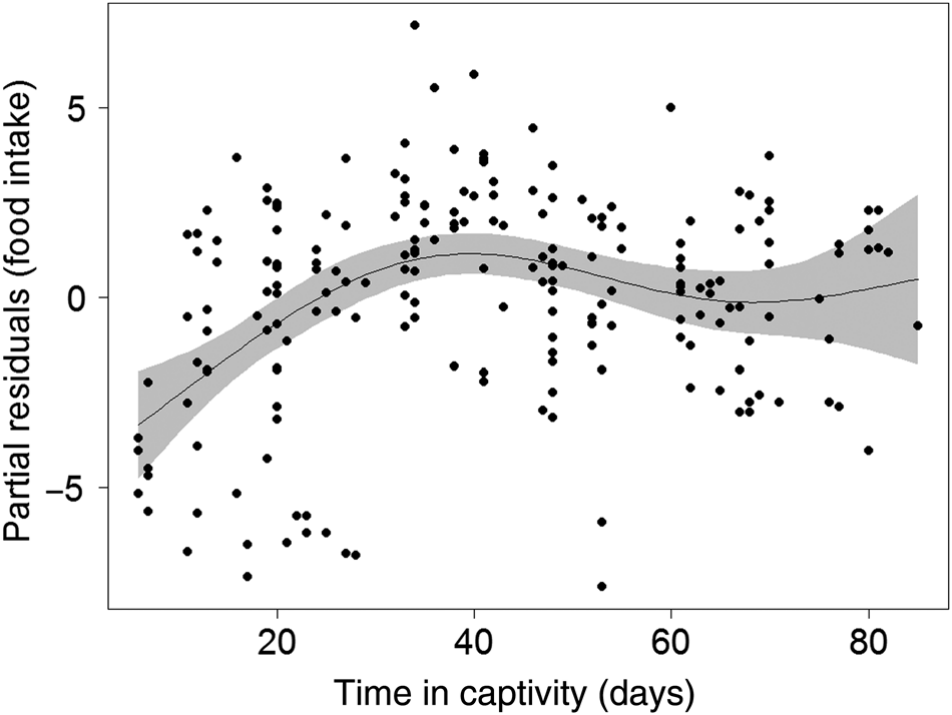
Partial residuals plot showing generalized additive mixed model-predicted changes in 3 day food intake with respect to time in captivity (black, continuous line). The 95% confidence limits for smoothed estimated effects are shown in grey.

The GAMM results indicated that 3 day food intake for juvenile sea lion was not significantly different 1–15 days after branding ($\hat{\beta }$ = 0.66, *t* = 0.96, *P* = 0.34) in comparison to periods before branding and, therefore, *SinceBrand* was removed from the model. In the resulting model, food intake changed in a non-linear manner during time in captivity (edf = 4.08, *F* = 8.27, *P* < 0.001), with a tendency to increase for the first 40 days, slightly decline until 60 days, and then flatten for the remainder of the time in captivity (Fig. 1). Intake was significantly lower during the period 1–15 days after LHX implant ($\hat{\beta }$ = −1.94, *t* = −2.82, *P* = 0.005) and then returned to initial levels (16–30 days, *t* = −1.01, *P* = 0.31; >31 days, *t* = 0.96, *P* = 0.34).

### Principal components analysis

The total variance in the 17 analytes within our data set was explained using six principal components with eigenvalues > 1 (Table 2). Based on communality scores, over one-half of the variance ($\hat{C}$ > 0.50) was explained in all analytes using six PCs, with the exception of potassium ($\hat{C}$ = 42%). Total variance explained by the PCA was highest for analytes GLOB, TP and RBC ($\hat{C}$ = 93, 86 and 84%, respectively). Analytes found to be strongly associated with each PC (loading scores > 0.40) were grouped and are shown with their respective loading values in Table 2. For example, WBC (loading score = −0.73) decreased as PC2 and other strongly associated analytes (calcium, albumin and creatinine) increased. All 17 analytes were found to be associated strongly with at least one PC, while potassium, haemoglobin and amylase were found to be associated with two PCs. Total bilirubin was found to be the only analyte associated strongly with PC6, while all other PCs had more than one strongly associated analyte. Based on having the highest loading score among strongly associated analytes within each PC, the following principal analytes were chosen: RBC (PC1, loading score = 0.90), WBC (PC2, −0.73), GLOB (PC3, 0.89), PLT (PC4, 0.72), GLU (PC5, 0.67) and TBIL (PC6, 0.77). The WBC was chosen over ALB as the principal analyte of PC2 based on unrounded loading scores of −0.728 and 0.725, respectively.

**Table 2:** Loading and communality scores ($\hat{C}$) for six principal components found to describe the variance in values for 17 blood sample analytes obtained from juvenile Steller sea lions during time in captivity

Blood analyte	Principal component loading scores						
	PC1	PC2	PC3	PC4	PC5	PC6	$\hat{C}$
Red blood cell count^a^	**0.90**	0.05	−0.01	−0.09	0.00	0.12	0.84
Haematocrit^b^	0.85	−0.07	0.11	0.12	0.03	0.16	0.78
Blood urea nitrogen	0.70	0.11	0.21	−0.01	0.15	−0.29	0.65
White blood cell count^a^	0.09	−**0.73**	0.10	0.13	−0.02	0.06	0.57
Albumin	0.30	0.73	−0.31	0.03	0.04	0.13	0.73
Calcium	−0.12	0.70	0.11	0.39	−0.06	−0.09	0.68
Creatinine	0.23	0.56	0.23	−0.35	0.12	0.24	0.61
Globulins	0.18	−0.26	**0.89**	0.05	−0.11	−0.14	0.93
Total protein	0.39	0.14	0.81	0.07	−0.10	−0.09	0.86
Alkaline phosphatase^a^	0.06	0.07	−0.64	0.17	0.08	−0.35	0.58
Potassium	0.22	−0.08	−0.43	0.06	−0.41	−0.12	0.42
Platelet count^a^	0.00	−0.12	−0.07	**0.72**	−0.17	−0.22	0.62
Haemoglobin	0.23	0.03	0.03	0.55	0.06	0.42	0.53
Amylase^a^	−0.38	0.27	−0.01	0.49	0.44	−0.06	0.65
Glucose	0.17	0.15	−0.08	−0.20	**0.67**	−0.02	0.55
Alanine amino transferase^a^	−0.09	0.36	0.19	−0.12	−0.61	0.03	0.56
Total bilirubin	0.03	0.03	0.03	−0.09	−0.01	**0.77**	0.61
Percentage of variance explained	27.5	16.6	21.4	13.8	11.0	9.7	
Eigenvalue	3.08	1.86	2.40	1.54	1.23	1.08	

Blood analytes with loading scores >0.40 or <−0.40 within a principal component were considered highly associated with that component and are outlined in red. The analyte found to be most highly correlated with each component (i.e. principal analyte; shown in bold) was modelled against factors potentially influencing the physiology of sea lions during their time in captivity. ^a^Logarithmically transformed. ^b^Arcsine square-root transformed.

### Factors influencing principal analytes

**Figure 2:**
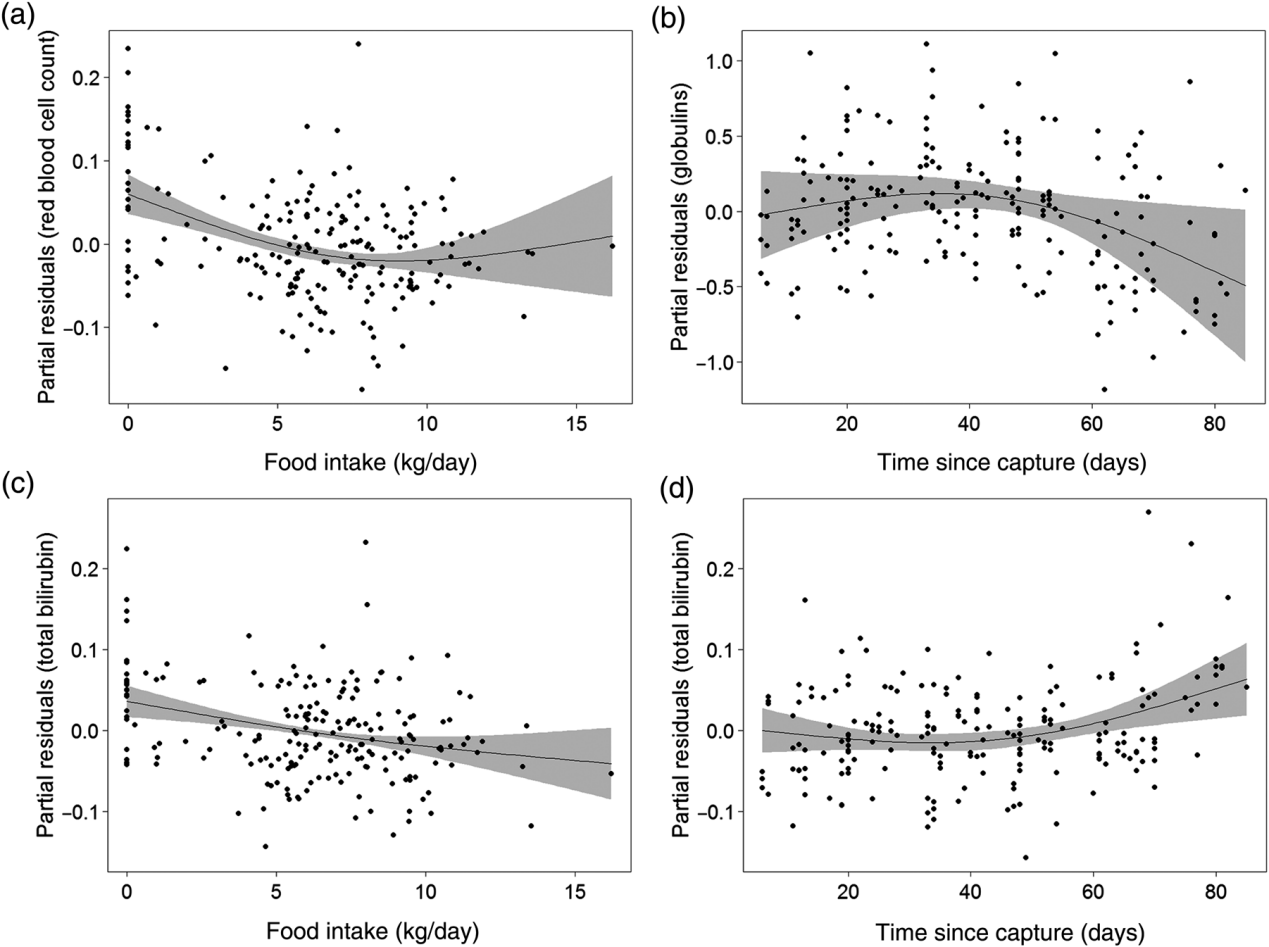
Partial residuals plots for principal analyte models. (**a**) Predicted response of red blood cell counts (log, 10^6^/mm^3^) with 3 day average food intake (in kilograms per day). (**b**) Changes in globulins (in grams per decilitre) with time in captivity. (**c**) Changes in total bilirubin (in milligrams per decilitre) with 3 day average food intake (in kilograms per day). (**d**) Changes in total bilirubin with time in captivity. The 95% confidence limits for the generalized additive mixed smoothed response (black line) are shaded in grey.

The model-averaging results, with the influence of predictor variables on each principal analyte, are presented in Table 3. The random intercept standard deviation (${\hat{\sigma }}_{\mathrm{RE}}$) is shown to provide a measure of the amount of analyte variance observed between individual sea lions. The global model for RBC was highly significant (log-likelihood ratio, LR = 93.68, *P* < 0.001). Significant model-averaged parameter estimates indicated that there was an influence of food intake and *SinceImplant* on RBC, which decreased until average food consumption reached ∼9 kg/day (Fig. 2a). The 95% confidence intervals for estimated RBC above 9 kg/day suggest that RBC was not influenced by higher food intake levels (grey region in Fig. 2a). The RBC was also found to be significantly higher 1–15 days after implant surgery and then lower during the following interval, 16–30 days. The WBC was strongly influenced by all predictor variables (LR = 85.73, *P* < 0.001). Associations with both *tCaptive* and food intake were reanalysed as linear (edf = 1), with WBC decreasing with time in captivity and food intake. The WBC remained elevated at 1–15 and 16–30 days post-branding and 1–15 days post-LHX implant surgery. The GLOB model was significant when including all predictor variables (LR = 106.77, *P* < 0.001). This included significant non-linear changes in GLOB in response to *tCaptive* and a positive linear response from food intake. Globulin was relatively constant with *tCaptive* until ∼38 days and then decreased over time (Fig. 2b). Furthermore, this analyte was elevated 1–15 days post-branding and during 1–15 and 16–30 days post-LHX implant surgery. A significant decrease in GLOB occurred 30–45 days post-branding but, given that there was no significant effect found 16–30 days post-branding, the importance of this effect is uncertain. The global PLT model was found to be significant (LR = 29.43, *P* = 0.002), but the best-fit model included only *SinceBrand*. This analyte was found to be elevated at 1–15 days post-brand, but returned to normal during subsequent periods. The global model for GLU was found to be non-significant (LR = 12.35, *P* = 0.34), suggesting that changes in this analyte (and the variation described in PC5) were not explained by any of variables we examined. Finally, the global model for TBIL was significant (LR = 46.89, *P* < 0.001) and was best fitted with non-linear smoother functions for *tCaptive* and food intake and the factor *SinceBrand*. Partial residual plots show that TBIL exhibited a marginally non-linear decline (edf = 1.34) with food intake that, based on confidence intervals, became non-significant at higher food consumption levels (Fig. 2c). Additionally, TBIL remained relatively constant for the first 40 days in captivity but then increased (Fig. 2d). Branding appeared to have a delayed effect on TBIL because there was no effect 1–15 days post-brand but a significant and increasingly negative effect during subsequent periods (16–30, 31–45 and >45 days).

**Table 3:** Parameter estimates and standard errors (in parentheses) for terms evaluated as predictors for each principal analyte

Analyte	${\hat{\sigma }}_{\mathrm{RE}}$	fx(time captive)	fx(food intake)	Time since brand (days)				Time since LHX implant (days)			Adjusted *r*^2^	LR	*P*-value
				1–15	16–30	31–45	>45	1–15	16–30	>30			
RBC	0.05	0.0026, 0.011 (0.010, 0.0093)	−0.056**, −0.012 (0.021, 0.0075)	−0.013(0.017)	−0.017(0.018)	0.014(0.022)	0.032(0.021)	0.090***(0.16)	−0.062**(0.20)	−0.056(0.35)	0.25	93.68	<0.001
WBC	0.13	−0.0056**(0.0018)	−0.016**(0.0054)	0.29***(0.049)	0.14*(0.059)	0.027(0.085)	0.091(0.099)	0.27***(0.057)	0.11(0.072)	0.059(0.11)	0.28	85.73	<0.001
GLOB	0.54	0.41*, −0.12(0.17, 0.092)	0.036**(0.012)	0.47***(0.11)	−0.091(0.13)	−0.33*(0.16)	−0.032(0.22)	0.75***(0.12)	0.68***(0.16)	0.50(0.27)	0.25	106.77	<0.001
PLT	0.18	0.043, −0.062(0.058, 0.043)	0.099, 0.012(0.066, 0.025)	0.17**(0.060)	−0.026(0.069)	0.026(0.088)	−0.065(0.099)	0.11(0.070)	−0.033(0.093)	−0.0024(0.15)	0.03	29.43	0.002
TBIL	0.02	−0.052*, 0.018*(0.022, 0.0088)	−0.0071, −0.17**(0.010, 0.0054)	−0.025(0.014)	−0.040**(0.015)	−0.048*(0.020)	−0.096**(0.030)	0.0067(0.017)	−0.015(0.024)	−0.030(0.040)	0.20	46.89	<0.0001

Results were produced for each analyte by second-order Akaike information criterion model-averaging of all candidate models. Principal analytes (response variables) included red blood cell count (RBC, log), white blood cell count (WBC, log), globulins (GLOB), platelets (PLT, log) and total bilirubin (TBIL). The model for glucose was found not to be significant; therefore, it is not shown. Parameters estimates and errors for two-coefficient smoother functions are separated by a comma. Results include the estimated standard deviation for animals' specific random intercepts (${\hat{\sigma }}_{\mathrm{RE}}$), adjusted *r*^2^ for the best-fit model with non-significant terms removed, likelihood ratio (LR) of the global (all terms) model vs. the null (random effect only) model, and significance of the global model (*P*-value). The level significance for each parameter is indicated as follows: *P < 0.05, **P < 0.01 and ***P < 0.001.

Values for principal analytes obtained at the time of capture were within the confidence range predicted for moderate to high levels of food intake, except for TBIL, which was within the ranges for all levels of food intake (Table 4). Also, no values were within the ranges predicted for trauma similar to branding or implant surgery. This result was expected based on the fact that only sea lions deemed healthy were included for temporary captive study.

**Table 4:** A comparison of mean values for principal analytes at time of capture to model predictions at various levels of food consumption and 1–15 days after branding and LHX implant surgery, assuming time of capture (*tCaptive* = 0)

Principal analyte	Mean value at capture	Food intake (predicted with no trauma)			1–15 days post-exposure (predicted with moderate food intake)	
		Fasted	Moderate	High	Brand	Implant
RBC (10^6^/mm^3^)	3.8	4.2 [4.1, 4.4]	4.0 [3.9, 4.0]	3.9 [3.8, 4.0]	n.s.	4.4 [4.2, 4.5]
WBC (10^3^/mm^3^)	10.2	12.6 [11.3, 13.9]	11.4 [10.3, 12.6]	10.9 [9.7, 12.2]	15.1 [13.2, 17.3]	14.9 [12.5, 17.7]
GLOB (g/dl)	3.5	3.6 [3.3, 3.9]	3.8 [3.5, 4.1]	3.9 [3.6, 4.3]	4.3 [3.9, 4.7]	4.6 [4.2, 5.0]
PLT (10^3^/mm^3^)	383	*n.s.*	414 [382, 448]	n.s.	488 [438, 544]	n.s.
TBIL (mg/dl)	0.35	0.38 [0.35, 0.41]	0.34 [0.31, 0.38]	0.33 [0.29, 0.37]	n.s.	n.s.

The 95% confidence intervals are shown in square brackets. Principal analytes were red blood cell count (RBC), white blood cell count (WBC), globulins (GLOB), platelets (PLT) and total bilirubin (TBIL). Food intake rates were 0 (fasted), 6.4 (moderate rate) and 9.3 kg/day (high rate) with no trauma. Effects of branding and LHX implant surgery 1–15 days post-exposure were estimated assuming a moderate food intake rate. Values predicted to be statistically the same as the moderate food intake rate are denoted ‘n.s.’.

## Discussion

For a wide range of vertebrates, haematology and clinical chemistry panels provide the starting point for diagnoses of general health, nutrition and disease state for wildlife biologists and veterinarians alike. While no single parameter can typically describe a condition exclusively, pattern recognition among variables is essential to stepwise determination of the most probable status (Kahn and Line, 2010). Most blood parameters can be associated with numerous physiological functions and are complicated in many species by variations with age, sex and geographical location (e.g. Hasselmeier *et al*., 2008; Greig *et al*., 2010; Clarke *et al*., 2013; Maas *et al*., 2013; Robert and Schwanz, 2013). While extensive reference ranges and elaborate metabolite tracking for a given disease state may exist for domestic species, the comparably limited information for many wild species at risk poses significant challenges for effective monitoring protocols and management strategies. In many instances, available reference data are restricted to small samples sizes from individuals in long-term captivity. However, when appropriate baseline data can be obtained, the possibilities, particularly for managed species, are wide ranging, such as monitoring the health of wild lions (Maas *et al.*, 2013), assessing parasite load and habitat quality in wallabies (Robert and Schwanz, 2013), assessing the efficacy of translocation in possums (Clarke *et al*., 2013) and determining the impact of eco-tourism on stingrays (Semeniuk *et al*., 2009).

Specific to pinnipeds, blood panels provide the backbone of rehabilitation treatment and monitoring and have been assessed for their power to predict survival probability (Greig *et al*., 2010; Witte *et al.*, 2014). Clinical biochemistry and haematology have been applied to field studies of prey switching in harbour seals (Thompson *et al*., 1997) and determining potential physiological differences between genetically distinct populations with opposite trajectories in Steller sea lions (Zenteno-Savin *et al*., 1997; Rea *et al*., 1998; Lander *et al.*, 2013). Interpretation of results from blood panels is greatly improved when age- and species-specific baselines and ranges are available. We used our varied data set to establish which parameters typically included in pinniped health assessments tend to shift in concert and compare that with what is known for other terrestrial and marine mammals. The magnitude of these shifts in response to varying levels of stressor may also help us to simplify and refine field and captive diagnostic approaches.

### Nutrition

Before any analysis, we recognized that nutrition can greatly impact most physiological parameters, and perhaps more so for this juvenile age class. Pinnipeds have been shown to differ statistically in haematology and clinical chemistry values with age, in particular during the pup to juvenile transition; however, chemistry values tend to be more robust (e.g. Mellish *et al*., 2006; Greig *et al*., 2010). Given that all of our animals were in the >12 month age class, we did not test for the effect of age, nor did we test for the effect of body condition (e.g. Greig *et al*., 2010). Juvenile Steller sea lions gradually increased their food intake until day 38 of time in captivity, contrary to a previously described 5 day fast after entry into captivity (Mellish *et al*., 2006). Some individuals maintained lower rates of daily food intake for greater periods of time (Fig. 1). This is likely to be due to a gradual adjustment to the captive environment, atypical daily recurrent foraging opportunities and the transition to consumption of fresh-thawed rather than live food. A slight decline in appetite after 40 days may have resulted from animals reducing their intake after reaching or exceeding the ideal weight for their size. A decline in appetite following LHX implant surgery persisted for up to 15 days, which corresponded to the period when sea lions appear to experience discomfort following surgery (Walker *et al.*, 2009).

### Principal analytes

We established six PCs that corresponded to different physiological changes experienced by sea lions while they were in captivity. One pitfall of the use of blood parameters for health assessments is that evaluations can be superficial and certain conditions may be overlooked if values of routinely measured parameters fall within larger established ‘normal’ variations (Vinkler *et al.*, 2010). Our PCA approach provided us with an objective way to establish which parameters were the best indicators of change. As an alternative to modelling the PCs themselves, we chose to model the principal analytes for each PC. It is important to note that the other dominant blood analytes associated with each PC would be expected to produce similar but not exactly the same results if they were modelled because they each contain slightly different types of physiological information. Our method, however, offered the important advantages of reducing the chance for reporting spurious effects, reducing redundancy for the reported effects and identifying key analytes for measuring changes in physiology resulting from changes in food intake, time in captivity and trauma.

### Hydration

Principal component 1, with a primary analyte of RBC, served as a primary indicator of hydration state, because decreases in blood volume are known to cause increases in both RBC and HCT. Furthermore, as indicated by changes in RBC with food intake (Fig. 2a), this effect diminished at moderate food intake levels when water intake probably exceeded output. A distinct increase in RBC immediately after LHX implant surgery aligns with the reduction in food intake observed 1–15 days after the procedure. Although it has been reported previously that Steller sea lion BUN concentrations may increase or decrease in response to fasting (Rea *et al*., 2000, 2009), we found a positive association between BUN and RBC (as indicated by component loading scores in PC1; Table 2). This may have been caused by the concentrating effects of dehydration during periods of food reduction without fasting.

### Inflammation

The primary analyte of PC2, WBC, behaved as anticipated for the primary immunological role of this variable. The WBC proved to be a good general indicator of both external tissue trauma (up to 16–30 days post-brand) and acute injury via surgical procedures (to 15 days post-implant). Our current larger sample size than previous studies and ability to control for the significant effects of food intake and time in captivity show that the inflammatory response caused by branding persists for as long as 3–4 weeks, which is longer than previously suggested. Inflammation caused by implant surgery, however, persisted for only ≤15 days. These physiological data are consistent with our visual observations that the surface wounding caused by branding could take >30 days to heal (Mellish *et al.*, 2007a), while the surgical incision sites generally appeared healed in <3 weeks (Mellish *et al*., 2007b). The WBC showed a negative change in response to food intake, which may have been the result of WBC concentrating during dehydration when fasting. A negative, linear change in WBC over the course of captivity may have been the result of overall decreases in parasite load, but we did not document parasites in this study. Another strong association with PC2 was ALB, a protein heavily involved in assisting other molecules (e.g. TBIL, CA) that is sensitive to dehydration and is often related to liver and kidney function. Noted increases in ALB were coincident with decreases in CA, suggestive of consumption of CA at the site of inflammation with WBC aggregation.

### Proteins

The third principal component was strongly and positively associated with GLOB, which is categorized as a general indicator of immune response. Elevated GLOB values during the first 15 days after branding and up to 30 days after surgical implant further support this interpretation. Values for GLOB at 31–45 days suggested that inflammatory immunoglobin production had subsided at this later stage and may have been further depressed by brand exudation, which has been shown to result in a hypoglobulin condition (Duncan *et al.*, 1994). Total protein was also highly associated with PC3, suggesting that changes in this parameter were highly associated or possibly driven by changes in GLOB. Although ALP is affected by a number of factors, including age, in marine mammals (Fair *et al.*, 2006), low values can indicate illness or malnutrition (Fothergill *et al*., 1991). Our observed variability in GLOB over time suggests that decreases in ALP may be related to a specific immune response, rather than the previous suggestion that it simply increases during captivity (Mellish *et al*., 2006).

### Tissue repair

Mammalian platelets are well known for their role in the clotting process and repair function. Increased PLT are expected whenever there is increased demand due to increased consumption of this particular blood component. Accordingly, PLT remained elevated during the first 15 days following the branding treatment, similar to what has been demonstrated in the healing process of human burn victims (Pavić and Milevoj, 2007). In contrast, the more acute, localized trauma of surgical implantation did not result in significant shifts in PLT (Mellish *et al.*, 2007b).

### Blood sugar

Perhaps not surprisingly, changes in GLU were not found to be attributed to any factors we examined. While it is a standard measure for monitoring human diabetes and can be associated with liver disease, trauma or parasitism in other terrestrial mammals, marine mammals have been shown to maintain stable blood GLU regardless of nutritional state (Rea *et al.*, 2009).

### Blood turnover

Principal component 6 was characterized by haemoglobin and its metabolite, TBIL. Total bilirubin can be increased during increased RBC destruction, fasting and liver disease in most mammals. In our case, TBIL declined as anticipated with *tCaptive* and increased food intake without a concurrent change in RBC (PC1).

### Varied response to injury

The physiological response of sea lions, as measured by blood analytes, was distinguishable between hot-iron branding and LHX surgical implantation. The RBC responded only to implant surgery, whereas PLT and TBIL responded only to branding. Both treatments had an effect on WBC and GLOB, but the duration of these effects differed. Increased RBC after implant surgery was probably an artifact of reduced food consumption and decreased hydration after surgery, rather than a direct physiological effect of the surgery. Changes in WBC were similar in magnitude between procedures (branding = 0.29 vs. implant = 0.27; Table 3), but the branding effect was still apparent at 16–30 days and lasted longer than previously reported (Mellish *et al*., 2007a). In contrast to WBC, GLOB effects were greater in magnitude ($\hat{\beta }$ = 0.75, 1–15 days) and longer lasting following implant surgery compared with branding ($\hat{\beta }$ = 0.47). This increase in GLOB was previously shown to be attributed largely to increases in haptoglobins, which peaked at ∼3 weeks post-surgery (Mellish *et al.*, 2007b). A response of PLT to branding has been reported previously, with mean values reaching a peak of 1100 m/mm^3^ at week 1 and then quickly subsiding by week 2 (Mellish *et al.*, 2007b). Our estimated peak value of 488 m/mm^3^ at 1–15 days (Table 4) incorporated data after the expected peak and may therefore have underestimated the effect of branding on PLT. As previously shown, PLT does not appear to have a strong response to implant surgery (Mellish *et al.*, 2007b) and was not impacted by food intake and time in captivity. Although TBIL showed changes with branding, this analyte may be less useful for evaluating the surface wounding because it appears to accumulate over a relatively long period and was found to be susceptible to the influences of food intake and time in captivity. Furthermore, most of the predicted changes in TBIL due to branding were below the level of sensitivity of our instrument (±0.1 mg/dl).

### Biological significance and application to field measures

We were able to discern varied responses to physiological challenges from a set of six principal analytes that are standard in most health-screening panels (RBC, WBC, GLOB, PLT, GLU and TBIL). The analytes responded as suggested by existing data for other large mammals. The RBC was sensitive to changes in hydration status. The WBC will reflect a similar level of response to varied traumas; however, the duration of elevation may be indicative of the type of physiological challenge. Globulin and PLT may serve as a good indicator of the clinical effects of surface wounding and may be useful for distinguishing those effects from deeper trauma. Glucose is relatively stable regardless of condition and may be useful only in diagnoses of conditions of extreme magnitude.

The values we predicted for blood parameters (Table 4) following trauma from branding and LHX implant surgery were all well within the range for what are considered representative for this age class (Bossart *et al*. 2001; Mellish *et al*., 2006). This suggests that while the changes we saw were significant, single-point data interpretation, as is typical in field collections, should be used with caution. Our values are most likely to be applied in a rehabilitation or captive monitoring situation. However, if there are large shifts in overall population values, our interpretation of major resultant parameter associations may be of use in formulating hypotheses regarding the underlying causes and aiding in directed research efforts.

## Funding

This work was supported by the Alaska SeaLife Center, North Pacific Research Board (R1011) and the Pollock Conservation Cooperative Research Center. This manuscript is referenced by the North Pacific Research Board as NPRB publication number 526.
